# Variability and Nativeness in the Mediterranean Taxa: Divergence and Phylogeography of *Genista etnensis* (Fabaceae) Inferred from Nuclear and Plastid Data

**DOI:** 10.3390/plants11223171

**Published:** 2022-11-19

**Authors:** Olga De Castro, Gianluigi Bacchetta, Salvatore Brullo, Emanuele Del Guacchio, Emanuela Di Iorio, Carole Piazza, Paolo Caputo

**Affiliations:** 1 Department of Biology, University of Naples Federico II, Botanical Garden, Via Foria 223, 80139 Naples, Italy; 2 Department of Life and Environmental Science, Conservation and Biodiversity Center (CCB), University of Cagliari, V.le Sant’ Ignazio da Laconi, 11-13, 09123 Cagliari, Italy; 3 Department Biological, Geological and Environmental Sciences, University of Catania, Via Antonino Longo 19, 95125 Catania, Italy; 4 National Botanical Conservatory of Corsica, Office of the Environment of Corsica, Avenue Jean Nicoli, 14, 20250 Corte, France

**Keywords:** mediterranean flora, molecular dating, nuclear DNA, plastid DNA, phylogeography, sanger sequencing

## Abstract

*Genista etnensis* is a remarkable and well-known tree endemic to Sicily, Sardinia, and Corsica (Mediterranean Basin). Nevertheless, its morphological variability and its native status throughout its range need to be further investigated. In this study, we aim to clarify some aspects of this infraspecific variability by molecular means. Sequences of one nuclear and five plastid markers were analyzed under maximum parsimony by using TCS software. Plastid data were also time-calibrated under a Bayesian Inference framework. Plastid data revealed strong isolation between the populations from the Cyrno-Sardinian biogeographical province, which are also the most diverse and presumably the most archaic, and those from Sicily and Southern Italy (in this latter area, the species is naturalized). The calibration analysis indicates that the last common ancestor between *G. etnensis* and its sister group *G. fasselata* dates back to the middle Pliocene or slightly later, when sclerophyllous Mediterranean vegetation spread, whereas *G. etnensis* itself might have originated in the middle Pleistocene. The current, rather unusual distribution of *G. etnensis* could be explained by long-range seed dispersal from the western part of the range or by anthropogenic introduction into Sicily, with extinctions of transported haplotypes in the region of origin. Interestingly, the Vesuvius population, introduced from Sicily in recent times and locally naturalized, shows private genotypes, and was richer in both genotypes and haplotypes than the Sicilian ones.

## 1. Introduction

The genus *Genista* L. (Fabaceae, Genisteae) includes about 150 species and over 180 taxa, including subspecies [1], of spiny or harmless shrubs (rarely small trees) (see [2] for a detailed morphological description). It is widespread from the Euro-Mediterranean region, where the genus is greatly diversified, with over than 120 species [3], to western Asia [4]. A greater concentration can be observed in the central and western sectors of the Mediterranean Basin (e.g., [2,5,6,7,8]). As known, its basin is one of the richest mega hot spots of plant biodiversity worldwide [9,10,11,12], and numerous taxa are narrow endemic and often limited to islands [13,14]. In various cases, distributions show disjunctions that are suggestive of past tectonic or climatic events [9,15,16,17,18,19,20]. This scenario has been reported for several *Genista* species [8,21], but, at least in *G. anglica* L. s.l. [22] and in some representatives of the *G. ephedroides* DC. group [23], the distribution has been partly attributed to anthropogenic dispersal in protohistoric or historical times. The well-known arboreal broom *G. etnensis* (Raf.) DC. ([24], and the nomenclatural section therein, for the correct name of the taxon) shows one of the most intriguing native distributions within the genus, as it occurs only in the three largest islands of the Mediterranean: Sicily, Sardinia, and Corsica [3]. In Sicily, it was discovered on Mt. Etna in 1810 or before by Bivona ([25] p. 27, sub *Spartium*), where it grows on lavas up to 2000 m a.s.l.; but it had been introduced for forestry purposes on the Peloritani massif a long time ago [26] and later in some neighboring localities of the Nebrodi Massif and in Lachea (Cyclopean Isles) [27]. Moris [28] first observed *G. etnensis* in Sardinia, where it is known for several siliceous mounts of the central and northern sectors of the island (rarely descending to the basal plain along riverbeds) [29]. Only in 1961 was the species reported as present in Corsica [30], where it occurs very locally along the south-eastern coasts [31,32,33]. For a detailed distribution, see Table 1. In addition, this plant was first introduced into Campania (southern Italy), i.e., in Ischia Island, the Sorrentine Peninsula, and, especially, Vesuvius [34,35], where it has become invasive [36]. Nowadays, as a naturalized or casual alien, it is also reported for several other regions of Italy, from Emilia-Romagna to Calabria [37].

On the basis of ecological considerations, some authors [38] hypothesized that *G. etnensis* originated in Sardinia, and only later spread to Sicily. Indeed, Valsecchi [39] seemed to suggest that the plant had been introduced to the latter island, where apparently it occurs mainly in man-influenced habitats. Despite this, Spada et al. [40] regard *G. etnensis* as an archaic species characterized by a relic distribution both in Sardinia and Sicily; in this latter island, it may have survived on the relatively recent Mt. Etna on account of the peculiar activity of this volcano, which periodically regenerates its cacuminal vegetation. In Corsica, it was believed to be introduced (e.g., [31,41,42,43,44]), but this statement has been vigorously confuted by the most recent studies [33,45]. In addition to its atypical native range, the species shows a remarkable taxonomic distinctness, which is suggested also macroscopically by its arboreal habitus, which is very unusual within the genus. This feature inspired Spach ([46] p. 152) in publishing the new monotypic genus *Dendrospartum* Spach, including *D. etnense* (Raf.) Spach (“aetnense”), and also characterized by several other features, among which is the calyx shape. Immediately after, Presl ([47] p. 568) segregated *D. etnensis* s.l. into the new genus *Drymospartum*, including *D. purgans* (L.) C. Presl, *D. etnense* (Raf.) C. Presl (“aetnense”), and the new species *D. sardum* C. Presl, which included the Sardinian population described by Moris as *G. etnensis* (see below). Valsecchi [39] kept the species in *Genista*, but in the monotypic section *Aureospartum* Vals., belonging to the subgenus *Spartocarpus* Spach (see also [48] for the treatment under *Cytisanthus*). This assessment has been supported by molecular analyses [49,50], according to which *G. etnensis* falls in a distinct subclade of the *Genista* clade, with several autapomorphies. From a karyological viewpoint, *G. etnensis* populations also show an unusual chromosome complement in Sicily and Sardinia (2n = 52) [51,52]. Remarkably, the populations from Corsica show 2n = 54 ([43] p. 263), which, in turn, is atypical as well.

If the phylogenetic position of *G. etnenis* appears as well defined [50], this is less true about the variability of the species throughout its distributional range. Indeed, several authors reported morphological differences between the Sardinian populations and those of Sicily. As reported above, already Presl [45] proposed to separate the plants of Sardinia and Sicily into two species, on the basis of the measures of flowers and legumes. A similar opinion was expressed both by Spach and Walpers ([53] p. 220), who adopted the name *Dendrospartum sardum* (C.Presl) Walp. (“sardoum”) for the Sardinian plant. In addition, Rizzi Longo and Feoli Chiapella [54] emphasized that the Sicilian populations show relevant differences in the micro-morphology of pollen grains when compared those of Sardinia. As far as the populations from Corsica are concerned, they have been recently described as a distinct subspecies, i.e., subsp. *fraisseorum* Fridl., on the basis of morphological, cytological, and ecological data ([33] p. 77). The same author provided a new combination, at the same rank, for most of the Sardinian populations, namely, subsp. *sarda* (C. Presl) Fridl. ([33] p. 80), stating, however, that those occurring near the coast of this latter island belong to subsp. *fraisseorum*.

As investigations are urgently needed to clarify the variability and nativeness of *G. etnensis* throughout its whole range, in this work we propose population-level molecular analyses using Sanger sequencing of nuclear and plastid markers. Indeed, these latter analyses have proven useful when applied to other Mediterranean taxa (e.g., [23,55,56,57,58,59]). Populations of *G. etnensis* from representative localities from Sardinia, Corsica, and Sicily were sampled. To these plants, we added a population from Mt. Vesuvius, in Campania, in light of the fact that *G. etnensis* was introduced there at the beginning of the twentieth century directly from Sicily ([34] p. 58), in order to compare variation in this allochthonous population with that of the native Sicilian populations.

## 2. Materials and Methods

### 2.1. Sampling and DNA Extraction

Eighty-three individuals of *G. etnensis* (Table 1) were sampled from Sicily (two localities; codes B and F), Sardinia (three localities; codes G, L, and N), Corsica (three localities; codes P, S + Ss, and T), and Campania (one locality; code V). Plant material was collected from ten individuals per population, except for the populations from Solaro (T), for which only three individuals were found. In addition, the ten sampled individuals from the Solenzara area were collected from two locations (Solenzara, six individuals; road between Sari and Solenzara, four individuals), which were separately recorded (codes S and SS). For the investigations requiring outgroups, five individuals of *Genista fasselata* Decne. (Israel) were employed (code GF; Table 1); the choice of this outgroup was carried out according to unpublished results by P. Caputo, aiming a molecular phylogeny of several sections of the genus *Genista*. DNAs were extracted by using the GeneAll Exgene Plant SV kit (GeneAll Biotechnology), according to the manufacturer’s instructions.

### 2.2. Molecular Markers Selection

A preliminary investigation was carried out on two samples per population by using 42 plastid DNA markers (Pr) and one nuclear DNA marker (N) in the search of variable DNA traits (Supplementary File S1). PCR amplification sequence reactions conditions were optimized during this initial step of the research.

This screening allowed selection of six variable DNA regions (Table 2), namely, *trn*Q^(UUG)^–*psb*K intergenic spacer (labelled as Pr1), *trn*S^(UGA)^–*psb*Z intergenic spacer (Pr8), *trn*V^(UAC)^–*atp*E intergenic spacer (Pr18), *psb*A–*trn*H^(GUG)^ intergenic spacer (Pr38), and first *ycf*3 intron (Pr11) from plastid DNA, and Internal Transcribed Spacers 1 and 2 plus the intervening *5.8*S (N43) from the nuclear ribosomal DNA. Amplifications of the genomic DNAs were carried out by using Q5 Hot Start High-Fidelity DNA Polymerase (New England Biolabs); several recalcitrant samples were amplified by using AmpONE Fast Pfu DNA Polymerase (GeneAll Biotechnology), in all cases according to the manufacturer’s instructions. Amplified fragments were purified by using DNA Clean & Concentrator-5 kit (Zymo Research). Information on the employed molecular markers is reported in Table 2.

### 2.3. DNA Sequencing

DNA sequences were obtained through modified Sanger chemistry by using the Bright Dye Terminator (iCloning), according to the manufacturer’s specification, purified by using the BigDye XTerminator Purification Kit (Applied Biosystems, Thermo Fisher Scientific), and loaded onto a 3130 Genetic Analyzer (Applied Biosystems, Thermo Fisher Scientific). Electropherograms were analyzed by AB DNA Sequencing Analysis ver. 5.2 software (Applied Biosystems, Thermo Fisher Scientific), edited by employing Chromas lite ver. 2.6.6 software (Technelysium Pty Ltd., http://technelysium.com.au/?page_id=13; accessed on 1 May 2021), and assembled by using ChromasPro ver. 2.1.8 software (Technelysium Pty Ltd.), which was used to detect the mutations (single nucleotide polymorphisms (SNP) or length mutations), generating the corresponding haplotypes and genotypes. The edited sequences were aligned by using ClustalW [60], as implemented in Mega ver. 10.1.8 software [61]; the latter software generated the matrices employed for the further analyses. The resulting sequences were submitted to GenBank (*G. etnensis*: nrDNA, OP794491–OP794496; cpDNA, OP830512-OP830515, OP830517-OP830518, OP830520-OP830521, OP830523-OP830524, OP830526-OP830528, *G. fasselata*: nrDNA, OP794497–OP794498; cpDNA, OP830516, OP830519, OP830522, OP830525, OP830529- OP830530).

### 2.4. Data Analysis

A genealogical investigation of the haplotypes and genotypes was carried out under a maximum parsimony (MP) framework, by using TCS ver. 1.21 software [62]. Plastid DNA sequence data for the five markers (i.e., Pr1, Pr8, Pr11, Pr18, and Pr38; Table 2) were merged into a single dataset, which was analyzed separately from the nuclear DNA sequence data (N43).

**Table 2:** Information on the molecular markers employed.

Code	Locus	(Name) Primer Sequence 5′-3′	Ta (°C)	Ref.
cpDNA				
Pr1	*trn*Q^(UUG)^-*psb*K IGS	(F: trnQ-IGSR)^§^ ACC CGT TGC CTT ACC GCT TGG (R: psbK-IGSR)^§^ ATC GAA AAC TTG CAG CAG CTT G	58	1
Pr8	*trn*S^(UGA)^-*psb*Z IGS	(F: psbZ-IGS) AAT AGC CAA TTG AAA AGC (R: trnS_UGA-IGSR)^§^ ATC AAC CAC TCG GCC ATC	55	1
Pr11	*ycf*3 intron-1	(F: ycf3-E1F)^§^ CAT TTA CCT ATT ACA GAG ATG G (R: ycf3-E2R) TTC CGC GTA ATT TCC TTC	50	1
Pr18	*trn*V^(UAC)^-*atp*E IGS	(F: atpE-IGSF)^§^ AGT GAC ATT GAT CCR CAA GAA GC (R: trnV_UAC-E1R) GTG TAA ACG AGG TGC TCT AC	57	1
Pr38	*psb*A-*trn*H^(GUG)^ IGS	(F: psbA3f) GTT ATG CAT GAA CGT AAT GCT C (R: trnHf)^§^ CGC GCA TGG TGG ATT CAC AATCC	55	2, 3
nrDNA				
N43	ITS1+5.8S+ITS2	(F: JK14)^§^ GGA GAA GTC GTA ACA AGG TTT CCG (R: SN3)^§^ TTC GCT CGC CGT TAC TAA GGG	55	4, 5

IGS = intergenic spacer; F = forward primer; R = reverse primer; Ta = annealing temperature; ^§^ = primer used for sequencing; Ref. = reference, 1 = [63]; 2 = [64]; 3 = [65]; 4 = [66]; 5 = [67].

Mononucleotide repeats from the cpDNA sequences were excluded from this analysis, as they may produce artifacts owing to possible homoplasy in the polyN sequences [68,69,70]. Analyses were run treating indels as missing data. When analyzing only *G. etnensis* haplotypes/genotypes, the parsimony threshold was set at 99%, whereas when *G. fasselata* was used to polarize the data, the parsimony threshold was set at 95%.

Plastid DNA data, the only one showing a detectable phylogenetic signal, were also investigated and time-calibrated under a Bayesian Inference framework. In the analyses described below, the most likely substitution model was computed by using jModeltest ver. 2.1.7 software [71] and applying the Akaike Information Criterion [72]. Given the comparatively small variation of our sequences, we preferred not to partition data in order to avoid over parametrization. In the absence of known macrofossils in the lineage of our species (or of the entire sect. *Spartocarpus*), calibration information (i.e., dates) obtained by Fiz-Palacios and Valcárcel [73] were employed. To the cpDNA sequences from the said paper ([73]; Supplementary Materials, Figure S2a), which originated from De Castro et al. [49], one sequence of *G. fasselata* was added (GenBank n. AJ890986), for a total of 26 taxa. A time-calibrated Bayesian analysis was carried out by using Beast ver. 2.5.1 software [74], hypothesizing a relaxed lognormal clock and a calibrated Yule prior and the chains were run for 5,000,000 generations. The dates from Fiz-Palacios and Valcárcel [73] employed for this analysis included that for the ingroup used in that paper (9.06 Ma) and that for the clade, including *G. radiata* (L.) Scop. and *G. tyrrhena* Vals. Clade (6.07 Ma), both with a lognormal prior. The date recovered for the node *G. fasselata–G. etnensis* was employed for a further calibration attempt within *G. etnensis*. A final calibrated Bayesian analysis was then carried out on our *G. etnensis* dataset; i.e., all our *G. etnensis* samples and the five specimens of *G. fasselata* for all our cpDNA markers, namely, Pr1, Pr8, Pr11, Pr18, and Pr38; Table 2. The Bayesian analysis was run for 50,000,000 generations, hypothesizing a relaxed lognormal clock, with a coalescent skyline prior and a lognormal prior for the date recovered from the preliminary calibration. The resulting trees and their associate information, such as their Posterior Probability (PP), 95% Highest Posterior Density (95%HPD), Node ages (in Ma), were explored by using Tracer ver. 1.7.1 software [75].

## 3. Results

### 3.1. Plastid Sequences

**Figure 1:**
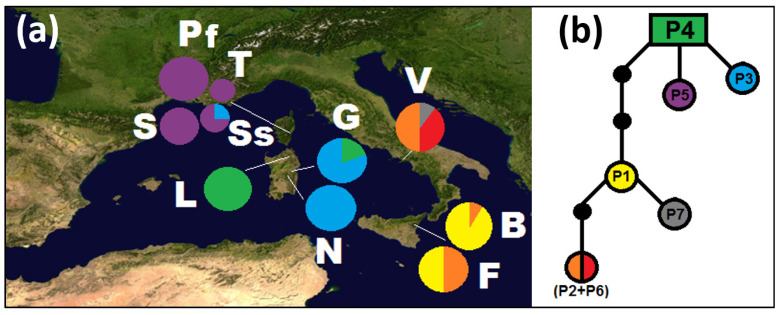
(**a**) Distribution map of the populations of *Genista etnensis* and the relative frequency of haplotypes (cpDNA) (populations labels: B = Mount Baracca, F = Fornazzo, G = Genna Silana, L = Mount Limbara, N = Villagrande Strisaili, Pf = Serra Fiumorbu, S = Solenzara, Ss = road between Sari and Solenzara, T = Solaro, V = Mount Vesuvius); (**b**) haplotype genealogies as inferred by TCS software (indels scored as missing data); black dots represent undetected haplotypes.

All the selected DNA sequences were polymorphic in *G. etnensis*. The point mutations and length variation in the cpDNA markers (Pr1, Pr8, Pr11, Pr18, and Pr38) yielded seven haplotypes (codes P1–P7; Table 3), when considering indels as a fifth state, or six, and scoring the indels as missing data (in this case, haplotype P6 from Vesuvius merges into haplotype P2). The length of the combined plastid dataset was of 1913 bp, with eight variable sites, of which five were parsimony-informative sites; considering the outgroup, the combined dataset was of 1929 bp with 30 variable sites of which 24 were parsimony-informative sites. In the outgroup two haplotypes were observed (GF_PA in three and GF_PB in two samples). The geographic distribution of the *G. etnensis* haplotypes is shown in Figure 1a. Each geographical region in which our sample is partitioned (i.e., Sardinia, Corsica, and Sicily/southern Italy) shows at least one regionally unique haplotype (Figure 1a), but only the Vesuvius population displays one (or two) private haplotype(s) in the strictest sense (P7, by considering the length mutations as missing data; P6 and P7, when scoring them as a fifth state).

The MP network of Figure 1b indicates close relationships between the Sicilian and continental southern Italian haplotypes, which are separated from the Cyrno-Sardinian ones; the latter are plesiomorphic in *G. etnensis*. In particular, when analyzing *G. etnensis* sequences only, the estimated outgroup haplotype is an exclusively Sardinian haplotype, P4, present in all the individuals at Mount Limbara and several individuals of Genna Silana (Figure 1a,b). When using *G. fasselata* as well, the haplotype directly connected to *G. fasselata* is P5, which is widespread in Corsica and absent from Sardinia.

### 3.2. Nuclear Sequences

**Figure 2:**
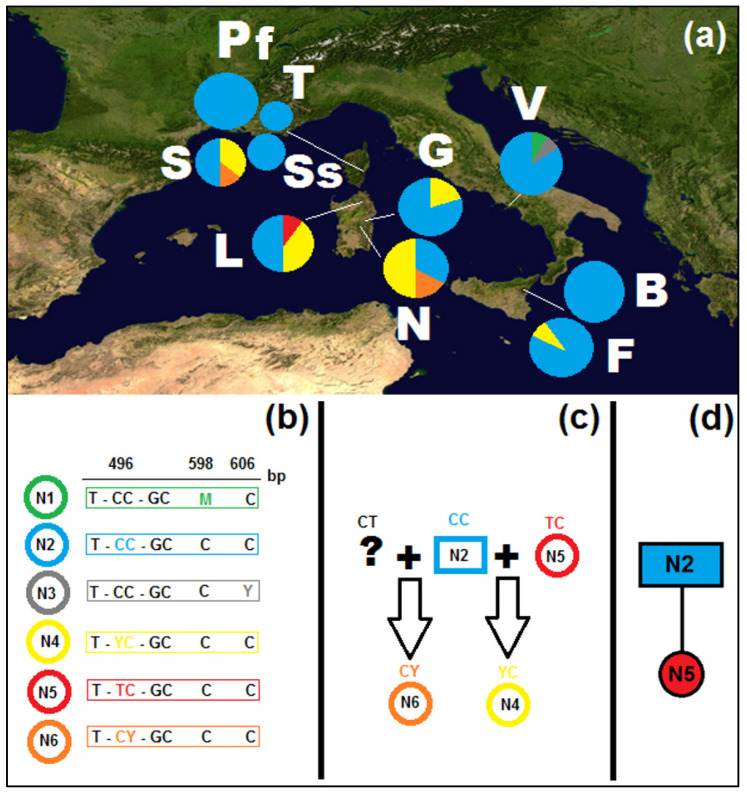
(**a**) Distribution map of the populations of *Genista etnensis* and relative frequency of the ITS genotypes (nrDNA) (populations labels: B = Mount Baracca, F = Fornazzo, G = Genna Silana, L = Mount Limbara, N = Villagrande Strisaili, Pf = Serra Fiumorbu, S = Solenzara, Ss = road between Sari and Solenzara, T = Solaro, V = Mount Vesuvius); (**b**) genotype and relative SNPs (see Supplemental File S2 for the alignment, GenBank accessions N1–N6: OP794491–OP794496); (**c**) possible crosses between genotypes; (**d**) reconstructed genealogy of unambiguous genotypes.

The nuclear ITS1, 5.8S, and ITS2 (650 bp) showed a lower genotypic variability, as compared to the cpDNA haplotype variation, and no length mutations (indels) were detected (Supplementary File S2). Six genotypes were detected (codes N1–N6; Figure 2a), one of which (N2) was widely distributed across all populations, with an overall frequency of 75% (from approx. 35% to 100% according to population). All mutations occur in the ITS2 region, either as individual mutations, as in the cases of genotypes N2 and N5, or as paralogues, in genotypes N1, N3, N4, and N6 (Figure 2b). Sardinia has the largest genotypic diversity, with four genotypes (N3, N4, N5, and N6), followed by Corsica, with three, represented also in Sardinia (N2, N4, and N6) and the Vesuvius area (N1, N2, and N3). In Sicily only two genotypes occur (N2 and N4). Private genotypes occur in Sardinia, in the Mount Limbara population (N5) and in the Vesuvius population (N1 and N3). The four detected paralogous genotypes may derive from crosses between unambiguous genotypes (e.g., see Figure 2c) and were not subjected to genealogical analysis.

In the outgroup, two genotypes were observed and correlated with the same specimens for the same haplotypes (GF_NA in three and GF_NB in two samples). The MP genealogy reconstruction for the two unambiguous genotypes (Figure 2d), both with and without the outgroup, indicates that the ancestral genotype is the widespread N2. As the investigated nuclear sequences included comparatively little information when compared to the cpDNA markers and given the paralogues did add a further measure of uncertainty, they were not further employed, and the following investigations were carried out exclusively on cpDNA sequences.

### 3.3. Molecular Dating

**Figure 3:**
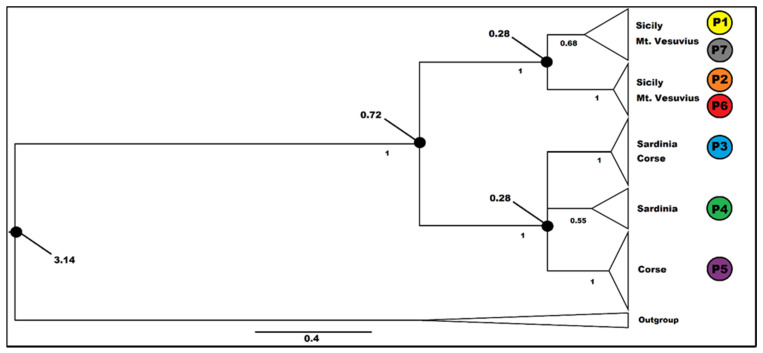
Calibrated Bayesian analysis on the plastid sequences of the inclusive group in the study of *Genista etnensis* populations (outgroup = *G. fasselata*). Posterior probabilities are indicated below the branches (PP > 0.5 are shown); relevant dates (Ma) on the nodes (black circle). Triangles are proportional to the number of individuals. For haplotype distribution and information, see Figure 1.

Bayesian calibration was carried out in two steps, as indicated in Materials and Methods. The best-fitting substitution model for the 26-terminal matrix for the preliminary first calibration on the literature data was the General Time Reversible (GTR) model. This investigation yielded a phylogram (data not shown) in which *G. fasselata* is a sister group to *G. etnensis*. The date for this sister group relationship is 3.365 MA (p.p = 0.935; 1.051-5.530 MA 95% HPD). This latter date was employed for the final calibration, including all 83 cpDNA sequences produced in this work for *G. etnensis* and the sequences of *G. fasselata*. Both the results of the preliminary and the final Bayesian analyses were convergent and all Estimated Sample Sizes were >> 100. The investigation on all the samples of *G. etnensis* recovered this taxon as monophyletic (PP = 0.9987), with a date of 0.718 MA (0.2157-1.3827 95% HPD). The species is divided into two major groups, one including all Sicilian and southern Italian samples (PP = 0.9967), with a date of 0.278 MA, and the other one including the Sardinian and Corsican samples (PP = 0.997), with a date of 0.277 MA. In both groups, several clades with high posterior probability can be observed (Figure 3), although no one corresponds to a single population. The only subgroup associated to a single geographic area is the one associated to the Corsican haplotype, P5.

## 4. Discussion

Biogeographically, *Genista etnensis* is an element of endemic to the Italo-Tyrrhenian superprovince [76,77]. The analysis of cpDNA data indicates strong isolation between the populations belonging to the province Cyrno-Sardinian province [77] and those from Sicily and Southern Italy; no haplotype, in fact, is shared between the two areas (Figure 1). Stronger affinities are on the contrary observed within the two lineages, with one haplotype shared between Sardinia and Corsica, and another between Sicily and Southern Italy. Sardinia and Corsica are home to three different haplotypes, Sicily to two and the Vesuvius area to three. The haplotype genealogy analysis carried out with TCS, when employing only the *G. etnensis* samples, reconstructs haplotype P4, which is unique to Sardinia, as plesiomorphic.

According to this pattern of descent, the populations of *G. etnensis* located in the areas between Supramonte (the historical Sardinian subregion including the Genna Silana mountain pass) and southern Gallura, i.e., central to northern Sardinia, retain the most archaic molecular features. This area, however, may only tentatively be regarded as coincident with that of the origin of the living populations of *G. etnensis*, given the fact that, when polarizing our TCS analysis by using *G. fasselata*, the most archaic haplotype is indeed P5, which is exclusive to Corsica. However, haplotype P4 is also present in Corsica at Solenzara. For this reason, we regard the whole Cyrno-Sardinian area as the location of the most plesiomorphic living populations of *G. etnensis*.

The six genotypes detected in nuclear ITS and 5.8S sequences were less informative than plastid DNA given their little variation, distribution, and the presence of paralogous sites, in addition to the possible problems related to incomplete concerted evolution when dealing with such sequences (e.g., [78]). However, differently from plastid DNA, the most widespread genotype (N2) is ubiquitous, and the second most frequent one (N4) is present in Corsica, Sardinia, and Sicily, being absent only from the Vesuvius area (Figure 2). The largest genotypic diversity is present in the Cyrno-Sardinian biogeographical province, with four genotypes (N2, N4, N5, and N6), three of which are present in both islands (N5 being represented only in the Limbara population of Sardinia); Sicily accounts for genotypes N2 and N4, both present also in Corsica and Sardinia. The Vesuvius area, on the contrary, in addition to the ubiquitous N2 genotype, has two private ones (N1 and N3). Overall, only two genotypes are unambiguous (N2 and N5); one (N4) may be accounted as a consequence of a cross between N2 and N5 and the other three as a consequence of crosses with unsampled or extinct genotypes.

The median date recovered in the calibration analysis for the sister group relationship between *G. etnensis* and *G. fasselata* (3.365 MA; 1.051–5.530 MA 95% HPD; Figure 3) indicates that their last common ancestor dates back to the middle Pliocene, roughly at the time of the appearance of the Mediterranean climate and its associated precipitation rhythm [79], or immediately after its onset, when a great reduction in subtropical taxa and a corresponding increase in sclerophyllous vegetation took place [79,80]. The age in which present-day *G. etnensis* populations started their diversification is 0.718 MA. Even when considering the interval of Highest Posterior Density (0.2157–1.3827 MA), the majority of this time interval is included in the so called “mid-Pleistocene revolution”, and its median value is almost perfectly coincident with the early-middle Pleistocene transition [81], when the first major glacial episode had just occurred, and a conspicuous fraction of the Tertiary subtropical flora had become finally extinct from Italy [82,83]. Justifying the present distribution pattern of *G. etnensis* is difficult. In fact, the range of *G. etnensis* is rather uncommon. By perusing the regional distribution of the taxa of the Italian Flora ([37], Appendix 2), only about ten species or subspecies are exclusively native to Sicily and Sardinia, constituting a rather disparaged assemblage of annuals or short-lived perennials, some of which have potential for epizoochorous or anemochorous long-range seed dispersal, the record in Sardinia for at least one of which (i.e., *Ranunculus pratensis* C. Presl) may be the result of confusion (see [84] p. 230). If we include also neighboring continental Southern Italy, only a few more species are added.

The Cyrno-Sardinian plate did not share a general tectonic history in common with Sicily [16,85,86], barring the existence of relationships between Sardinia, Kabilie (Algeria), and the Calabrian-Peloritan arc, much earlier than the proposed time frame for the origin of *G. etnensis* (see, for example, [87]). In addition, eustatic sea level variations, given the depth of the sea surrounding Sardinia and Corsica, only justify the biogeographic connections between these islands and maritime Tuscany, or more generally north-western Italy [16,88]. However, in spite of the great geologic and biogeographic separation between the Cyrno-Sardinian and Sicilian provinces [77], Schmitt et al. [88], in a comprehensive study based on over 100 research papers dealing with animals and plants, recovered definite biogeographic relationships between the two regions [88]. These relationships, however, are likely a consequence of the distribution of vertebrates more than plant species.

In light of the above, the present-day distribution of our species does not depend upon vicariance. Dispersal, however, calls into question a possible mechanism and a direction. For the first issue, no information is available for *G. etnensis* (barring a vague reference to heath-mediated pod explosion by Paiero et al., [89] p. 160) and the paucity in literature on the genus indicates that probably myrmecochory is involved in at least *G. tinctoria* L. [90]; a report is also available that non-native ants gather seeds from two species of the related *Teline* Medik. In the Iberian Peninsula [91], barochory is also suggested for an unidentified species of *Genista* from Pantelleria island [27]. Therefore, no long-range dispersal mechanism is known. Indeed, an event of exceptional, naturally occurring long-range dispersal may be envisaged (albeit unlikely). Given the dates recovered for the two main groups into which *G. etnensis* samples are divided (slightly over 250 thousand years for both), anthropogenic dispersal would be ruled out, unless our calibration data are incorrect as a consequence of an unusually fast mutation rate (which, in particular, would easily justify the different haplotypic pattern of the plants from Vesuvius). Nevertheless, without hypothesizing a fast mutation rate, a recent, man-mediated dispersal event may be hypothesized, by supposing that introduced haplotypes became extinct in the regions of origin. The direction of such an event is easier to envisage, as said above; our TCS analyses would indicate either a Sardinian or a Corsican haplotype as plesiomorphic and, therefore, our species of interest dispersed from the Cyrno-Sardinian province to Sicily. In this regard, we recall that Sicily and Sardinia have a long, if not intense, history of economic and social contacts which, within the limits of written history, date back to the times in which Carthaginians fought against Greeks for the control of Sicily and kept coastal colonies in Sardinia, from roughly the seventh century BCE and until the Punic wars (e.g., [92]). In addition, this would not be the only species of genus *Genista* for which recent anthropogenic dispersal has been hypothesized to justify unusual distributions. In fact, the existence of *G. cilentina* Vals., endemic to the southern coasts of Campania, has been regarded as the consequence of a deliberate introduction from a population of *G. numidica* Spach from Algeria [93,94]. In the same way, the *Genista* of the island Mezzu Mare (Ajaccio gulf, Corsica) is not a Corsican microendemic species (*G. mezzumarensis* Coulot & Rabaute), as assumed by Coulot and Rabaute ([95] pp. 786–793). An investigation conducted by Paradis and Chiappe [96], within the local population, showed that it is *G. tyrrhena* Vals. subsp. *pontiana* Brullo & De Marco. A few individuals were collected by a fisherman, born on Ponza Island (Pontine Islands, Latium, Italy) and living in Corsica, and were then planted in Ajaccio around 1982 or 1984, and whose seeds were sown on Mezzu Mare Island, probably in April 1987. The expansion of this broom on the island, initially very slow, seems to have become rapid from 2003 [97]. This example of voluntary introduction followed by rapid progression of a non-indigenous taxon on a given territory illustrates once again the importance of the human factor for understanding the chorology of many plant entities. In the light of the comparative homogeneity of haplotypes within the two areas (three very similar haplotypes in the Sardinian and Corsican areas and two for Sicily), the variation found in the Vesuvius area, with one (or two) private haplotypes, is indeed puzzling, given that the population is, as was already said, a consequence of purposeful anthropogenic dispersal in very recent times. Moreover, this population is distinct not only from a molecular but also from a morphological point of view (in flowers, legumes, and seeds; S. Brullo, unpublished data). At present, is would be very difficult to understand whether variation has increased in the new environment, with plants undergoing rapid diversification, or whether the plants that now are found in the Vesuvius area originate from an unsampled (or extinct) haplotype from Sicily.

## Figures and Tables

**Table 1:** Investigated samples of *Genista etnensis* and the outgroup.

Code	Taxon	Locality		N
B	*G. etnensis* (Raf.) DC.	Sicily	Mount Baracca	10
F	*G. etnensis*	Sicily	Fornazzo	10
G	*G. etnensis*	Sardinia	Genna Silana	10
L	*G. etnensis*	Sardinia	Mount Limbara	10
N	*G. etnensis*	Sardinia	Villagrande Strisaili	10
Pf	*G. etnensis*	Corsica	Serra Fiumorbu	10
S + Ss	*G. etnensis*	Corsica	Solenzara + road between Sari and Solenzara	6 + 4
T	*G. etnensis*	Corsica	Solaro	3
V	*G. etnensis*	Southern Italy	Mount Vesuvius	10
GF	*G. fasselata* Decne.	Israel	Mount Carmel	5

**N** = number of individuals sampled per population.

**Table 3:** Mutations in the five selected cpDNA markers and corresponding haplotypes in *Genista etnensis* populations. See also Table 1 for the molecular markers’ information and Figure 2 for the haplotypes’ geographic distribution.

Pr1 *			GenBank	Pr8 *	GenBank	Pr11 *	GenBank	Pr18 *		GenBank	Pr38 *				GenBank	H	Pop(n)
213	214	320		11		332		149	349		112	188	189	190			
T	G	---	OP830512	---	OP830517	C	OP830520	A	T	OP830523	AATAAGA	A	G	A	OP830526	P1	B(9), F(5)
T	G	---		---		C		C	G	OP830524	---	A	G	A	OP830527	P2	B(1), F(5), V(5)
T	G	AATTATACATATATATAA	OP830513	---		T	OP830521	A	T		---	T	C	T	OP830528	P3	G(8), N(10), Ss(1)
T	G	---		---		C		A	T		---	T	C	T		P4	G(2), L(10)
G	G	---	OP830514	---		C		A	T		---	T	C	T		P5	P(10), S(6), Ss(3), T(3)
T	G	---		TATAC	OP830518	C		C	G		---	A	G	A		P6	V(4)
T	T	---	OP830515	---		C		A	T		---	A	G	A		P7	V(1)

* = the base position corresponds to the sequence of the B1 sample, as referenced in Supplementary Files S3–S7; --- = deletion; H = haplotype; Pop = population; n = samples that have the haplotype.

## Data Availability

The datasets generated in the current study are available from the corresponding author on reasonable request.

## Supplementary Materials

The following supporting information can be downloaded at: https://www.mdpi.com/article/10.3390/plants11223171/s1, Supplementary File S1: Information and results on the molecular markers tested (+ = variable sequence; 0 = non-variable sequence; x = amplified with abnormal structure; − = low amplification efficiency) (Pr = plastid DNA; N = nuclear DNA); Supplementary File S2: Alignment of N43 molecular marker (ITS) for all dataset; Supplementary File S3: Alignment of Pr1 molecular marker (*trn*Q^(UUG)^–*psb*K IGS) for all dataset; Supplementary File S4: Alignment of Pr8 molecular marker (*trn*S^(UGA)^–*psb*Z IGS) for all dataset; Supplementary File S5: Alignment of Pr11 molecular marker (*ycf*3 intron 1) for all dataset; Supplementary File S6: Alignment of Pr18 molecular marker (*trn*V^(UAC)^–*atp*E IGS) for all dataset; Supplementary File S7: Alignment of Pr38 molecular marker (*psb*A–*trn*H^(GUG)^ IGS) for all dataset. Refs. [49,63,98,99,100,101] have been cited in supplementary materials.
