# Supplementary material for: Variability and Nativeness in the Mediterranean Taxa: Divergence and Phylogeography of Genista etnensis (Fabaceae) Inferred from Nuclear and Plastid Data
Source: Plants (Basel). 2022 Nov 19;11(22):3171. doi: 10.3390/plants11223171 (PMC9698455; doi:10.3390/plants11223171)
Supplement: Supplementary file 1 [file plants-11-03171-s001.zip › Supp_Files_v3/Sup_File_S1_Markers tested.pdf]

Supplementary Information for

Variability and nativeness in the Mediterranean taxa: divergence and phylogeography of *Genista etnensis* (Fabaceae) inferred from nuclear and plastid data.

Olga De Castro[1], Gianluigi Bacchetta[2], Salvatore Brullo[3], Emanuele Del Guacchio[1*], Emanuela Di Iorio[1], Carole Piazza[4], Paolo Caputo[1]

Supplementary File S1.

Table S1. Information and results on the molecular markers tested (+ = variable sequence; 0 = non-variable sequence; x = amplified with abnormal structure; - = low amplification efficiency) (Pr = plastid DNA; N = nuclear DNA).

**Cod.**	**Marcatore**	**Ref.**	**Note**	**Cod.**	**Marcatore**	**Ref.**	**Note**
**Pr1**	*trn*Q$^{(UUG)}$-*psb*K IGS	1	+	**Pr23**	*ycf*4-*ycf*10 IGS	1	0
**Pr2**	*psb*K-*trn*S$^{(GCU)}$ IGS	1	x	**Pr24**	*pet*A-*psb*J IGS	1	-
**Pr3**	*trn*G$^{(UCC)}$-*atp*A IGS	1	+	**Pr25**	*pet*L-*psa*J IGS	1	-
**Pr4**	*atp*F intron	1	0	**Pr26**	*psa*J-*rpL*20 IGS	1	x
**Pr5**	*atp*F-*atp*H IGS	1	0	**Pr27**	*rpL*20-*rp*S12 IGS	1	-
**Pr6**	*atp*H-*atp*I IGS	1	-	**Pr28**	*clp*P intron 2	1	-
**Pr7**	*rpo*C1 intron	1	x	**Pr29**	*clp*P intron 1	1	-
**Pr8**	*trn*S$^{(UGA)}$-*psb*Z IGS	1	+	**Pr30**	*clp*P-*psb*B IGS	1	0
**Pr9**	*psa*A-*ycf*3 IGS	1	+	**Pr31**	*psb*H-*pet*B IGS	1	-
**Pr10**	*ycf*3 intron 2	1	+	**Pr32**	*pet*B-*pet*D IGS	1	-
**Pr11**	*ycf*3 intron 1	1	+	**Pr33**	*rp*S3-*rp*S19 IGS	1	0
**Pr12**	*ycf*3-*trn*S$^{(GGA)}$ IGS	1	-	**Pr34**	*ccs*A-*ndh*D IGS	1	x
**Pr13-14**	*rp*S4-*trn*T$^{(UGU)}$_ver.1	1	+	**Pr35**	*psa*C-*ndh*E IGS	1	0
**Pr15**	*trn*F$^{(GAA)}$-*ndh*J IGS	1	+	**Pr36**	*ndh*E-*ndh*I IGS	1	x
**Pr16**	*ndh*C-*trn*V$^{(UAC)}$ IGS	1	-	**Pr37**	*rp*S15-*ycf*1 IGS	1	x
**Pr17**	*trn*V$^{(UAC)}$ intron	1	0	**Pr38**	*psb*A-*trn*H$^{(GUG)}$ IGS	2	+
**Pr18**	*trn*V$^{(UAC)}$-*atp*E IGS	1	+	**Pr39**	*trn*Q$^{(UUG)}$-*rps*16 IGS	3	0
**Pr19**	*atp*B-*rbc*L IGS	1	0	**Pr40**	*trn*L$^{(UAA)}$ intron	4	0
**Pr20**	*rbc*L-*acc*D IGS	1	x	**Pr41**	*trn*L$^{(UAA)}$-*trn*F$^{(GAA)}$ IGS	4	0
**Pr21**	*acc*D-*psa*I IGS	1	-	**Pr42**	*trn*G$^{(UCC)}$-*trn*S$^{(GCU)}$ IGS	5	+
**Pr22**	*psa*I-*ycf*4 IGS	1	0	**N43**	ITS1-ITS2	6	+

References

1 Prince, L.M. Plastid Primers for Angiosperm Phylogenetics and Phylogeography. *Appl. Plant Sci.* **2015**, *3*, 1400085. https://doi.org/10.3732/apps.1400085.

2 Kress, W.J.; Erickson, D.L. A Two-Locus Global DNA Barcode for Land Plants: The Coding rbcL Gene Complements the Non-Coding *trn*H-*psb*A Spacer Region. *PLoS ONE* **2007**, *2*, e508. https://doi.org/10.1371/journal.pone.000050.
3 Calviño, C.I.; Downie, S.R. Circumscription and phylogeny of Apiaceae subfamily Saniculoideae based on chloroplast DNA sequences. *Mol. Phylogenet. Evol.* **2007**, *44*, 175-191. https://doi.org/10.1016/j.ympev.2007.01.002.
4 Taberlet, P.; Gielly, L.; Pautou, G.; Bouvet, J. Universal primers for amplification of three non-coding regions of chloroplast DNA. *Plant Mol. Biol.* **1991**, *17*, 1105-1109. https://doi.org/10.1007/BF00037152.
5 Hamilton, M.B. Four primer pairs for the amplification of chloroplast intergenic regions with intraspecific variation. *Mol. Ecol.* **1999**, *8*, 521-523.
6 De Castro, O.; Cozzolino, S.; Jury, S.L.; Caputo, P. Molecular relationships in *Genista* L. Sect. *Spartocarpus* Spach (Fabaceae). *Plant Syst. Evol.* **2002**, *231*, 91-108. https://doi.org/10.1007/s006060200013.
